# Reply to the Letter to the editor “Ipragliflozin improves the hepatic outcomes of patients with diabetes with NAFLD”

**DOI:** 10.1002/hep4.1996

**Published:** 2022-05-09

**Authors:** Hirokazu Takahashi, Keizo Anzai

**Affiliations:** ^1^ Division of Metabolism and Endocrinology, Faculty of Medicine Saga University Saga Japan; ^2^ Liver Center, Saga University Hospital, Faculty of Medicine Saga University Saga Japan

We thank van Kleef et al. for their interest and discussion regarding our recent publication.^[^ ^1^ ^]^ Their letter pointed out three important issues: the low power because of the small sample size involved in testing the effect of ipragliflozin on hepatic outcome, low baseline γ‐glutamyl transpeptidase (GGT) levels of the control group that could result in their nonsignificant decrease, and the unmatched prevalence of dyslipidemia between the control group and intervention group. As stated in our publication,^[^ ^1^ ^]^ the small sample size, which was calculated to test the effect of ipragliflozin on the glycemic control of nonalcoholic fatty liver disease (NAFLD) with type 2 diabetes, was an obvious limitation to testing the liver‐related endpoint in subgroup analysis. This small sample size led to unmatched backgrounds, including baseline GGT levels and the prevalence of dyslipidemia between groups. Therefore, the effect of ipragliflozin on the NAFLD liver in our study should be interpreted with caution, as van Kleef et al. suggested.

Accumulating evidence indicates that sodium–glucose cotransporter 2 (SGLT2) inhibitors improve the abnormal liver enzymes and steatosis in NAFLD with type 2 diabetes.^[^ ^2^ ^]^ Therefore, SGLT2 inhibitors would be an expected therapeutic choice for NAFLD in patients with type 2 diabetes. However, glucagon‐like peptide‐1 receptor agonists (GLP‐1RA) are recognized as a treatment for nonalcoholic steatohepatitis with fibrosis stage 2 or 3 in diabetes,^[^ ^3^ ^]^ although the hepatoprotective effect of liraglutide has not been confirmed in patients with diabetes,^[^ ^4^ ^]^ and the dose used in the recent study with semaglutide is not currently available for prescription in patients with diabetes.^[^ ^5^ ^]^ Pioglitazone is a robust choice, but body weight gain is not permissible in many NAFLD cases.

To validate the effect of SGLT2 inhibitors on hepatic outcome in NAFLD with diabetes, further studies are needed in larger populations, as van Kleef et al. suggested. In particular, a pathological improvement should be confirmed. In the real‐world clinical setting and in the absence of optimal choices, SGLT2 inhibitors could be considered as a therapeutic choice for NAFLD with diabetes, as well as pioglitazone and regular‐dose GLP‐1RA for diabetes.

## CONFLICT OF INTEREST

K.A. received grants from Astellas Pharma. H.T. received grants from Abbvie, SISMEX, and Astellas.
